# Effectiveness of 577-nm Yellow Laser in the Management of Steroid-Induced Rosacea: A Prospective Study

**DOI:** 10.7759/cureus.112557

**Published:** 2026-07-13

**Authors:** Yasir Radhi, Hayder Alhamami

**Affiliations:** 1 Department of Dermatology, Al-Shaheed Al-Sadr General Hospital, Baghdad, IRQ; 2 Department of Dermatology, Iraqi Board for Medical Specialization, Baghdad, IRQ

**Keywords:** 577-nm yellow laser, facial erythema, laser therapy, steroid-induced rosacea, telangiectasia, vascular laser

## Abstract

Background: Steroid-induced rosacea (SIR) is a challenging condition resulting from prolonged topical corticosteroid use and is characterized by persistent erythema, telangiectasia, papules, and pustules. Although the 577-nm yellow laser has demonstrated efficacy in rosacea and other vascular dermatoses, its role in the treatment of SIR has not previously been investigated.

Objective: To evaluate the safety and short-term clinical outcomes of 577-nm yellow laser therapy in patients with steroid-induced rosacea and to assess treatment outcomes according to clinical and telangiectatic subtypes.

Methods: In this prospective study, 50 consecutive patients with steroid-induced rosacea were enrolled. Exclusion criteria included age <20 years, pregnancy, autoimmune disorders, and Fitzpatrick skin phototypes V-VI. Baseline assessment included eight symptoms and eleven clinical signs evaluated using standardized grading scales. Patients were treated with a 577-nm yellow laser (FlexSys®, GME GmbH, Germany) using a power of 20-23 W, pulse duration of 16-20 ms, and 50% coverage density. Clinical evaluations were performed before each treatment session and one month after the final session.

Results: The mean duration of topical corticosteroid use was 43.2 ± 36.95 months (range: 4-240 months). The erythematotelangiectatic subtype was the most common clinical presentation (38/50, 76%), followed by the erythematous subtype (8/50, 16%) and papulopustular subtype (4/50, 8%). Among patients with a classifiable telangiectatic morphology (n = 42), network-like vessels were the predominant telangiectatic pattern (28/42, 66.7%), followed by arborizing superficial capillaries (10/42, 23.8%) and blue deep venules (4/42, 9.5%). After a median of two treatment sessions (range: 1-7), significant clinical improvement was observed. The greatest improvement among symptoms was seen in stinging sensation (13/50, 26%; 85.8%), diffuse redness (43/50, 86%; 84.1%), and papulopustular nodules (8/50, 16%; 79.7%). Among clinical signs, erythema (47/50, 94%; 87.0%) and flushing (33/50, 66%; 79.3%) demonstrated the highest response rates. In contrast, dryness (32/50, 64%; 17.7%), xerosis (17/50, 34%; 23.5%), wrinkles (2/50, 4%; 27.8%), and comedones (9/50, 18%; 35.1%) showed more limited improvement. Among telangiectatic morphologies, network-like vessels exhibited the most favorable therapeutic response.

Conclusion: The findings of this prospective study suggest that a 577-nm yellow laser may represent a safe and promising adjunctive treatment for steroid-induced rosacea, particularly for improving erythema and superficial telangiectatic lesions when used as part of a standardized treatment protocol. To our knowledge, this is the first prospective study evaluating 577-nm yellow laser therapy in steroid-induced rosacea. Treatment outcomes appeared to vary according to telangiectatic morphology, with network-like vessels demonstrating more favorable responses than deeper vascular patterns. Larger controlled studies with longer follow-up are warranted to validate these findings.

## Introduction

Steroid-induced rosacea (SIR) is a rosacea-like facial dermatosis that develops following prolonged use of topical corticosteroids or after their withdrawal. Clinically, it resembles rosacea and is characterized by varying combinations of persistent erythema, papules, pustules, edema, and telangiectasia on a diffuse erythematous background [1].

Although the exact incidence of SIR remains unknown, it appears to affect women more frequently than men, likely reflecting the widespread use of topical corticosteroids for pigmentary disorders and cosmetic purposes. The condition is most commonly reported in middle-aged adults, although cases have been documented across all age groups [2,3].

Management of SIR remains challenging. The cornerstone of treatment is discontinuation of topical corticosteroids, frequently accompanied by a rebound inflammatory flare. Conventional management typically includes topical and systemic anti-inflammatory agents, particularly doxycycline and topical antimicrobials, with variable clinical outcomes [4].

The 577-nm yellow laser was originally developed for retinal vascular disorders and has subsequently been introduced into dermatologic practice for the treatment of vascular lesions and erythematous dermatoses [5,6]. Owing to its absorption peak by oxyhemoglobin, the 577-nm wavelength enables selective targeting of superficial cutaneous vessels while minimizing nonspecific thermal injury to surrounding tissues [6]. In addition, its monochromatic emission avoids the green wavelength component present in copper bromide lasers, potentially reducing epidermal melanin absorption and the risk of post-inflammatory hyperpigmentation [7].

Previous studies have demonstrated the efficacy of the 577-nm yellow laser in rosacea and other facial vascular disorders [4,6,7]. However, to the best of our knowledge, its role in steroid-induced rosacea has not been previously investigated. Furthermore, no study has evaluated whether treatment outcomes differ according to the dermoscopic morphology of telangiectatic vessels in SIR [8].

Therefore, this prospective study was conducted to evaluate the clinical outcomes and safety of 577-nm yellow laser therapy in patients with steroid-induced rosacea and to determine whether treatment response varies according to clinical and telangiectatic subtypes [9].

## Materials and methods

Study design and ethical approval

This prospective clinical study was conducted at a laser-equipped private dermatology clinic between January 2024 and January 2025. Ethical Approval was obtained from the Ethics Committee of the Iraqi Society of Dermatologists and Venereologists (ISDV-EC), Approval No. ISDV-EC/2023/YH-12-01 (December 15, 2023). The study was conducted in accordance with the principles of the Declaration of Helsinki. Written informed consent was obtained from all participants before enrollment.

Study population

**Figure 1:**
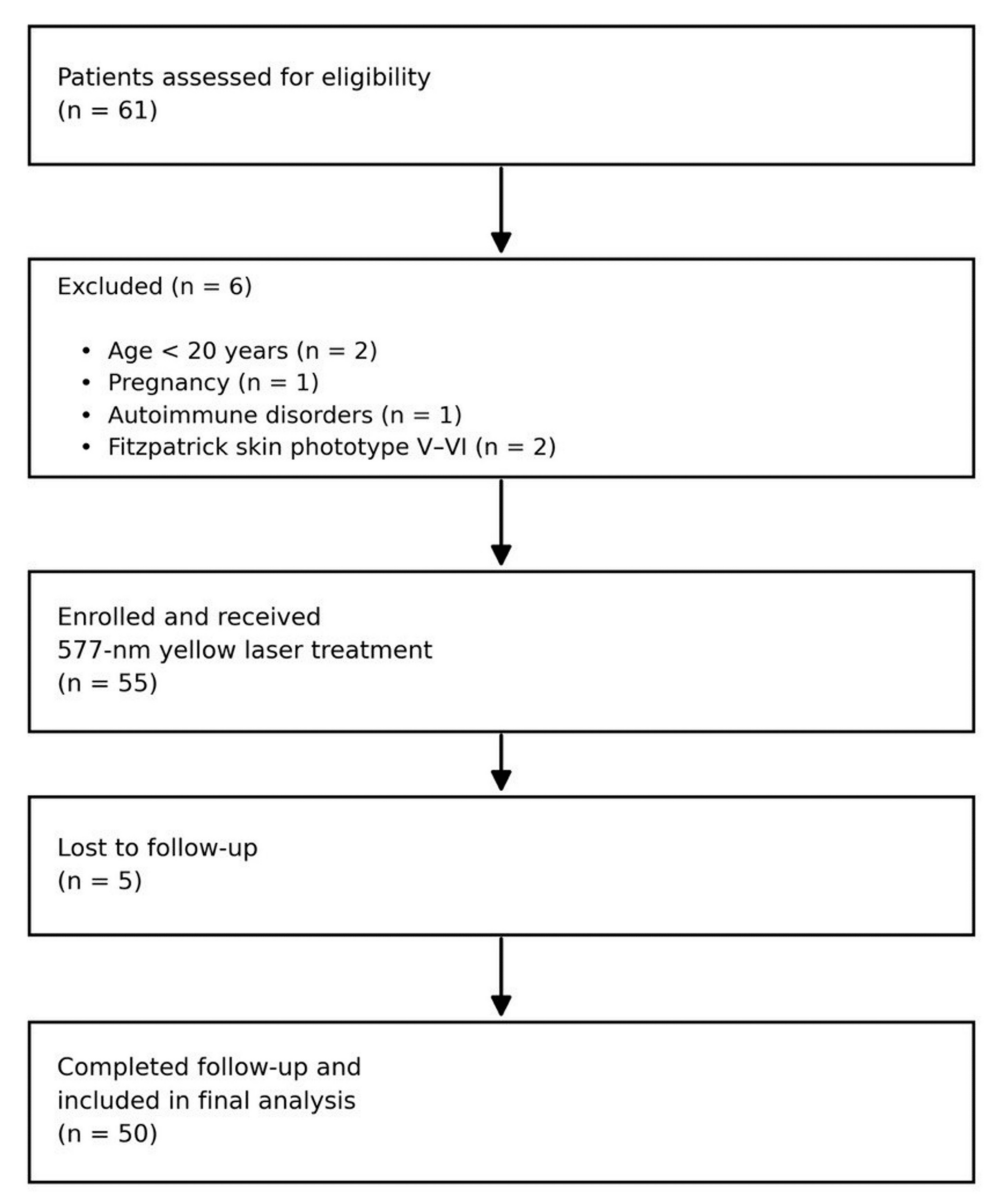
Study flow diagram illustrating patient selection, exclusions, enrollment, follow-up, and final analysis.

During the study period, 61 consecutive patients with clinically suspected steroid-induced rosacea (SIR) were assessed for eligibility. Patients younger than 20 years of age, pregnant women, individuals with autoimmune disorders, and those with Fitzpatrick skin phototypes V or VI were excluded. In addition, five enrolled patients did not complete follow-up and were excluded from the final analysis. The patient selection process is illustrated in Figure 1.

A total of 50 patients completed the study and were included in the final analysis. The diagnosis of SIR was established based on a history of topical corticosteroid use together with characteristic clinical findings, including persistent facial erythema, flushing, papules, pustules, and/or telangiectasia.

Topical corticosteroids were withdrawn using a standardized tapering protocol applied to all patients. Mometasone furoate was administered twice daily during the first week and once daily during the second week, followed by complete discontinuation. The tapering regimen was initiated at the first treatment visit and was identical for all participants. No systemic antibiotics, topical antimicrobials, topical calcineurin inhibitors, or other anti-inflammatory treatments were prescribed during the study period. Patients were permitted to use sunscreen only.

Treatment protocol

Before each laser session, patients thoroughly cleansed their faces to remove cosmetics, sunscreen, and other topical products using gentle cleansing or oil-free wipes. Treatment was performed using a 577-nm yellow laser system (FlexSys®, GME GmbH, Germany) with a power setting of 20-23 W, pulse duration of 16-20 ms, and coverage density of 50%.

Following the application of optical-grade cooling gel, a single treatment pass was delivered to the entire face while patients wore protective metal eye shields. The forehead was excluded from the treatment field because of minimal clinical involvement in most patients and the potential risk of post-treatment edema extending toward the periorbital region.

Immediately after treatment, the cooling gel was removed. Patients were instructed to avoid topical products for 12 hours and were counseled regarding expected transient adverse effects, including erythema (24-48 hours), edema (two to seven days), and microcrusting (five to seven days). Treatment courses consisted of one to seven sessions performed at four-week intervals according to individual clinical response.

Clinical evaluation

At baseline, patients completed a standardized questionnaire evaluating eight symptoms: diffuse redness, itching, dryness, burning sensation, skin tightness, papulopustular nodules, stinging, and photosensitivity. Each symptom was graded using a four-point scale (0 = absent, 1 = mild, 2 = moderate, and 3 = severe).

Clinical and dermoscopic examinations were performed to assess eleven signs: erythema, papulopustular rash, edema, comedones, telangiectasia, hirsutism, rebound phenomenon, xerosis, flushing, atrophy, and wrinkles. Each sign was graded using the same four-point scale.

Telangiectatic morphology was classified dermoscopically as network-like vessels, arborizing superficial capillaries, or deep blue venules.

Follow-up assessments were performed before each treatment session and one month after the final treatment session. Standardized digital photographs were obtained using an iPhone 15 Pro Max (48-megapixel camera) under consistent lighting conditions.

Outcome assessment

Therapeutic response for each symptom and sign was calculated using the following formula:

$\text{Improvement (\%)} = \frac{ \text{Baseline Score}-\text{Final Score}}{\text{Baseline Score} \times 100}$.

For each clinical variable, treatment outcomes were categorized as no response (<20% improvement), moderate response (20-49%), good response (50-74%), and very good response (≥75%).

For patient-level analyses, overall treatment response was defined as the mean percentage improvement across all evaluated parameters and was categorized as moderate (20-49%), good (50-74%), or very good (≥75%) improvement.

Statistical analysis

Statistical analyses were performed using SPSS version 25 (IBM Corp., Armonk, NY, USA). Categorical variables were presented as frequencies and percentages, whereas continuous variables were expressed as mean ± standard deviation (SD) or median (range), as appropriate.

Changes before and after treatment were assessed using the Wilcoxon matched-pairs signed-rank test. Comparisons between outcome groups were performed using the Kruskal-Wallis test for continuous variables and the Chi-square test or Fisher’s exact test for categorical variables, as appropriate. A p-value of <0.05 was considered statistically significant.

## Results

**Figure 2:**
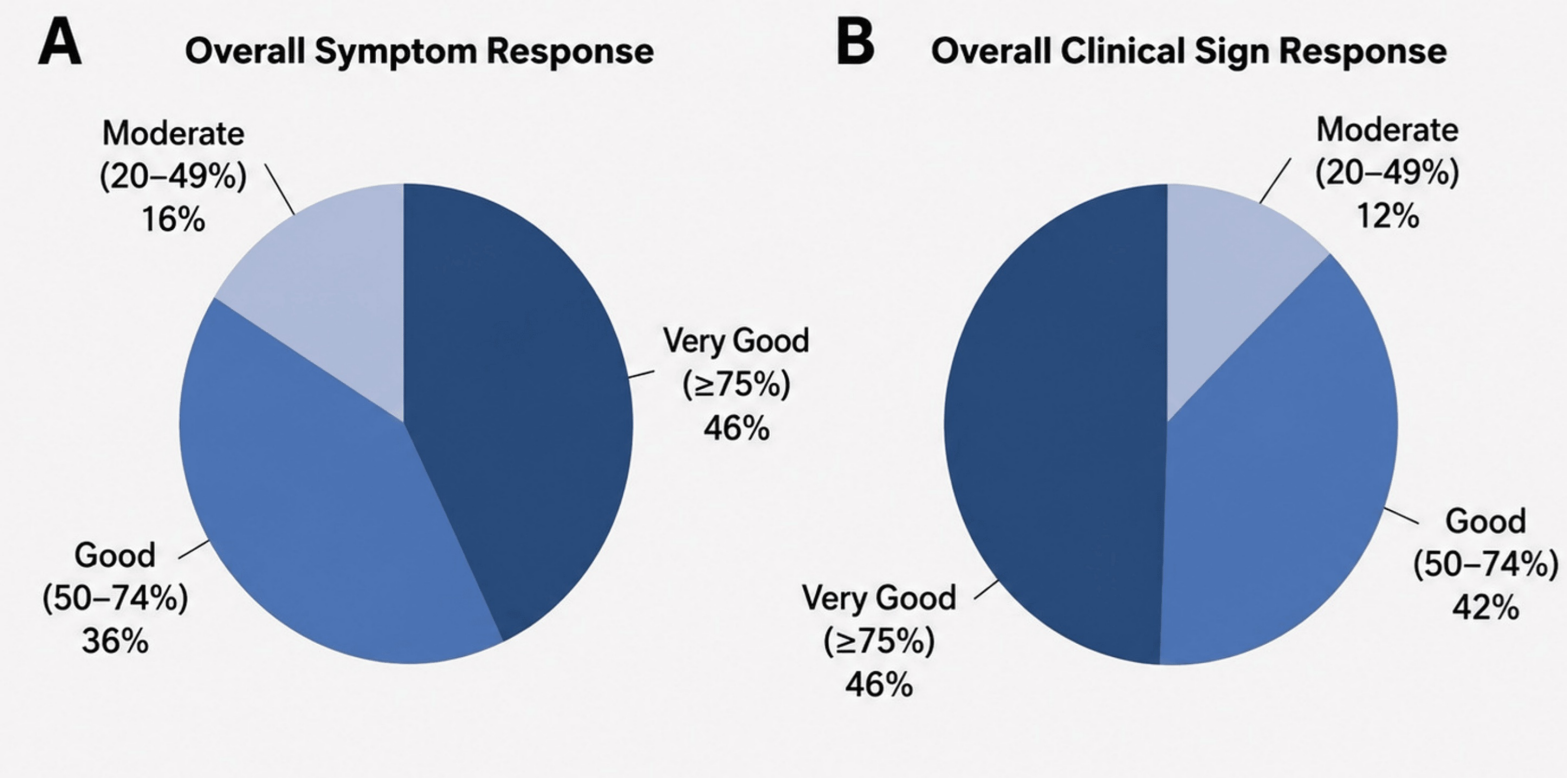
Overall treatment response for symptoms and clinical signs following 577-nm yellow laser therapy in patients with steroid-induced rosacea. (A) Overall symptom response. (B) Overall clinical sign response. Treatment outcomes were categorized as moderate (20-49% improvement), good (50-74% improvement), and very good (≥75% improvement). Percentages represent patient-level overall responses based on mean improvement scores.

A total of 61 patients were assessed for eligibility during the study period. Six patients were excluded because they did not meet the eligibility criteria, leaving 55 patients who were enrolled and received treatment. Five patients were subsequently lost to follow-up, resulting in 50 patients who completed the study and were included in the final analysis (Figure 2).

The mean age of the study population was 38 ± 7 years (range, 21-54 years), and the majority were female (48/50, 96%). As summarized in Table 1, melasma was the most common indication for topical corticosteroid use (28/50, 56%), followed by skin lightening (17/50, 34%). Khalta (combination preparations) was the most frequently used corticosteroid type (39/50, 78%). The mean duration of corticosteroid use was 43.2 ± 36.95 months (range: 4-240 months).

The erythematotelangiectatic subtype was the most common clinical presentation, accounting for 38 patients (76%), followed by the erythematous subtype in eight patients (16%) and the papulopustular subtype in four patients (8%). Among patients with a classifiable telangiectatic morphology (n = 42), network-like vessels represented the predominant dermoscopic subtype, observed in 28 patients (66.7%), whereas arborizing superficial capillaries and deep blue venules were identified in 10 (23.8%) and four patients (9.5%), respectively (Table 1).

**Table 1:** Demographic and clinical characteristics of patients with steroid-induced rosacea (n = 50). *Percentages for telangiectatic subtypes were calculated among patients with a classifiable telangiectatic morphology (n = 42). The three patterns were mutually exclusive and sum to 100%.

Variable	Category	n	%
Age (years)	Mean ± SD	38 ± 7	-
	Range	21-54	-
Sex	Female	48	96.0
	Male	2	4.0
Main indication for corticosteroid use	Melasma	28	56.0
	Skin lightening	17	34.0
	Allergy	3	6.0
	Rosacea	1	2.0
	Dryness	1	2.0
Type of corticosteroid	Khalta (combination preparation)	39	78.0
	Betamethasone	3	6.0
	Clobetasol	4	8.0
	Mometasone	4	8.0
Duration of corticosteroid use (months)	Mean ± SD	43.2 ± 36.95	-
	Range	4-240	-
Clinical subtype	Erythematous	8	16.0
	Papulopustular	4	8.0
	Erythematotelangiectatic	38	74.0
Telangiectatic subtype*	Network-like vessels	28	66.7
	Arborizing superficial capillaries	10	23.8
	Blue deep venules	4	9.5

Following a median of two laser sessions (range: 1-7), significant improvement was observed in both symptoms and clinical signs (Table 2). The greatest improvement among symptoms was observed for stinging sensation (13/50, 26%; 85.8%), diffuse redness (43/50, 86%; 84.1%), and papulopustular nodules (8/50, 16%; 79.7%). Among clinical signs, erythema (47/50, 94%; 87.0%) and flushing (33/50, 66%; 79.3%) demonstrated the highest response rates. In contrast, dryness (32/50, 64%; 17.7%), xerosis (17/50, 34%; 23.5%), wrinkles (2/50, 4%; 27.8%), and comedones (9/50, 18%; 35.1%) showed comparatively limited responses.

**Table 2:** Baseline and post-treatment symptom and sign scores following 577-nm yellow laser therapy. Values are presented as mean ± SD. n: number of patients in whom the variable was present at baseline; %: proportion of the total analyzed cohort (n = 50). Improvement (%) is the mean of individual patients’ percentage improvements, each calculated as ((baseline score-post-treatment score)/baseline score) × 100; these values therefore cannot be re-derived from the group mean scores shown above. P-values were obtained using the Wilcoxon matched-pairs signed-rank test. NA: not applicable (n = 1).

Variable	n	%	Baseline mean ± SD	Post-treatment mean ± SD	P-value	Improvement (%)
Symptoms						
Diffuse redness	43	86.0	2.77 ± 0.53	0.42 ± 0.66	<0.001	84.11
Itching	35	70.0	1.86 ± 0.77	0.83 ± 0.75	<0.001	60.48
Dryness	32	64.0	2.28 ± 0.68	1.84 ± 0.72	0.002	17.70
Burning sensation	24	48.0	2.08 ± 0.83	0.46 ± 0.59	<0.001	79.17
Skin tightness	14	28.0	1.36 ± 0.63	0.36 ± 0.50	0.001	71.31
Papulopustular nodules	8	16.0	2.13 ± 0.84	0.25 ± 0.46	0.011	79.74
Stinging	13	26.0	1.08 ± 0.28	0.07 ± 0.27	0.001	85.79
Photosensitivity	49	98.0	2.76 ± 0.43	0.61 ± 0.61	<0.001	77.22
Clinical signs						
Erythema	47	94.0	2.62 ± 0.68	0.33 ± 0.52	<0.001	87.00
Papulopustular rash	7	14.0	2.14 ± 0.38	0.88 ± 0.64	0.023	45.96
Edema	1	2.0	1.00	0.00	NA	100.00
Comedones	9	18.0	1.56 ± 0.53	0.89 ± 0.33	0.014	35.10
Telangiectasia	46	92.0	2.70 ± 0.51	0.67 ± 0.60	<0.001	73.92
Hirsutism	9	18.0	1.33 ± 0.50	0.30 ± 0.48	0.003	75.00
Rebound phenomenon	31	62.0	2.74 ± 0.51	0.85 ± 0.61	<0.001	66.68
Xerosis	17	34.0	2.35 ± 0.61	1.20 ± 0.96	0.002	23.52
Flushing	33	66.0	2.48 ± 0.67	0.43 ± 0.56	<0.001	79.30
Atrophy	6	12.0	1.83 ± 0.41	0.36 ± 0.50	0.014	57.14
Wrinkles	2	4.0	2.50 ± 0.71	1.50 ± 0.71	0.157	27.77

**Figure 3:**
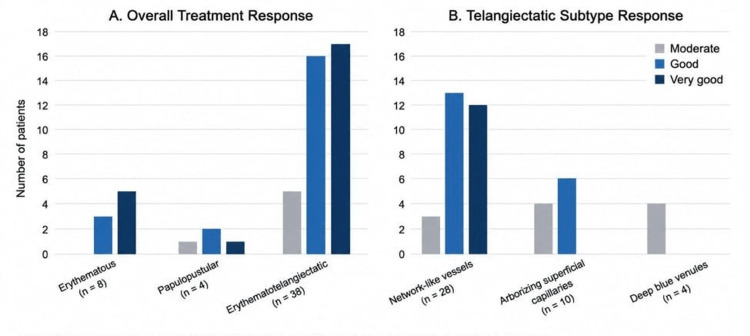
Treatment response according to clinical subtype and telangiectatic morphology in patients with steroid-induced rosacea. (A) Overall treatment response among the erythematous (n = 8), papulopustular (n = 4), and erythematotelangiectatic (n = 38) clinical subtypes. (B) Treatment response according to telangiectatic morphology within the erythematotelangiectatic subtype, including network-like vessels (n = 28), arborizing superficial capillaries (n = 10), and deep blue venules (n = 4). Network-like vessels demonstrated the most favorable therapeutic response, whereas deep blue venules showed only moderate improvement.

A very good overall response (≥75% improvement) was achieved in 24 patients (48%) for symptoms and 23 patients (46%) for clinical signs. Moderate improvement (20-49%) was observed in 8 patients (16%) for symptoms and in 6 patients (12%) for signs, respectively (Figure 3A).

Treatment outcomes according to individual clinical features are summarized in Table 3. Complete resolution (100% improvement) was most frequently observed for erythema (32/47, 68.1%), hirsutism (6/9, 66.7%), and flushing (18/33, 54.5%). In contrast, 7 of 17 patients with xerosis (41.2%) showed no response to treatment, while dryness and itching persisted in 19 of 32 (59.4%) and 5 of 35 patients (14.3%), respectively. All patients with telangiectasia demonstrated some degree of clinical improvement; however, the most common outcome category was a good response (50-74% improvement), observed in 24 of 46 affected patients (52.2%). Similarly, all patients with rebound phenomenon showed improvement, with 21 of 31 patients (67.7%) achieving a good response (Table 3).

**Table 3:** Distribution of treatment response outcomes for individual symptoms and signs following 577-nm yellow laser therapy. Response-category values are presented as numbers (%) calculated within each variable’s own n. The % column (after n) denotes the proportion of the total analyzed cohort (n = 50) in whom the variable was present at baseline. Response categories: no response (<20% improvement), moderate response (20-49%), good response (50-74%), and very good response (≥75% improvement).

Variable	n	%	No response n (%)	Moderate response (20-49%) n (%)	Good response (50-74%) n (%)	Very good response (≥75%) n (%)
Symptoms						
Diffuse redness	43	86.0	1 (2.3)	4 (9.3)	9 (20.9)	29 (67.4)
Itching	35	70.0	5 (14.3)	3 (8.6)	15 (42.9)	12 (34.3)
Dryness	32	64.0	19 (59.4)	6 (18.8)	7 (21.9)	0 (0.0)
Burning sensation	24	48.0	1 (4.2)	1 (4.2)	8 (33.3)	14 (58.3)
Skin tightness	14	28.0	2 (14.3)	0 (0.0)	3 (21.4)	9 (64.3)
Papulopustular nodules	8	16.0	0 (0.0)	0 (0.0)	2 (25.0)	6 (75.0)
Stinging	13	26.0	1 (7.7)	0 (0.0)	0 (0.0)	12 (92.3)
Photosensitivity	49	98.0	0 (0.0)	3 (6.1)	24 (49.0)	22 (44.9)
Clinical signs						
Erythema	47	94.0	0 (0.0)	1 (2.1)	14 (29.8)	32 (68.1)
Papulopustular rash	7	14.0	1 (14.3)	0 (0.0)	5 (71.4)	1 (14.3)
Edema	1	2.0	0 (0.0)	0 (0.0)	0 (0.0)	1 (100.0)
Comedones	9	18.0	3 (33.3)	0 (0.0)	5 (55.6)	1 (11.1)
Telangiectasia	46	92.0	0 (0.0)	7 (15.2)	24 (52.2)	15 (32.6)
Hirsutism	9	18.0	0 (0.0)	0 (0.0)	3 (33.3)	6 (66.7)
Rebound phenomenon	31	62.0	0 (0.0)	4 (12.9)	21 (67.7)	6 (19.4)
Xerosis	17	34.0	7 (41.2)	6 (35.3)	4 (23.5)	0 (0.0)
Flushing	33	66.0	1 (3.0)	1 (3.0)	13 (39.4)	18 (54.5)
Atrophy	6	12.0	0 (0.0)	0 (0.0)	5 (83.3)	1 (16.7)
Wrinkles	2	4.0	0 (0.0)	1 (50.0)	1 (50.0)	0 (0.0)

Association of treatment outcome with demographic and clinical features

No significant association was found between treatment outcome and age (p = 0.570), sex (p = 0.105), type of corticosteroid used (p = 0.919), duration of corticosteroid use (p = 0.368), or number of laser sessions (p = 0.479), as shown in Table 4 and Figure 3B.

**Table 4:** Association of treatment outcome with demographic and clinical characteristics. Data are n (%) unless otherwise stated; percentages are calculated within each row category. Overall patient-level response categories: moderate (20-49%), good (50-74%), and very good (≥75% mean improvement); no patient fell into the no-response (<20%) category. P-values: age and median (range) variables, Kruskal-Wallis test; categorical variables, Chi-square or Fisher's exact test.

Variable	Total (n = 50)	Moderate (n = 6)	Good (n = 21)	Very good (n = 23)	P-value
Age (years), mean ± SD	38 ± 7	34.5 ± 9	38 ± 8	39 ± 7	0.570
Sex, n (%)					0.105
Female	48 (96.0)	5 (10.4)	18 (37.5)	25 (52.1)	-
Male	2 (4.0)	1 (50.0)	1 (50.0)	0 (0.0)	-
Type of corticosteroid, n (%)					0.919
Khalta	39 (78.0)	5 (12.8)	16 (41.0)	18 (46.2)	-
Betamethasone	3 (6.0)	0 (0.0)	1 (33.3)	2 (66.7)	-
Clobetasol	4 (8.0)	1 (25.0)	1 (25.0)	2 (50.0)	-
Mometasone	4 (8.0)	0 (0.0)	1 (25.0)	3 (75.0)	-
Duration of corticosteroid use (months), median (range)	48 (4-240)	36 (12-60)	48 (6-84)	39 (22-54)	0.368
Number of laser sessions, median (range)	2 (1-7)	2 (1-3)	2 (1-4)	2 (1-7)	0.479
Clinical subtype, n (%)					
Erythematous	8 (16.0)	0 (0.0)	3 (37.5)	5 (62.5)	0.651
Papulopustular	4 (8.0)	1 (25.0)	2 (50.0)	1 (25.0)	0.337
Erythematotelangiectatic	38 (76.0)	5 (13.2)	16 (42.1)	17 (44.7)	1.000

**Figure 4:**
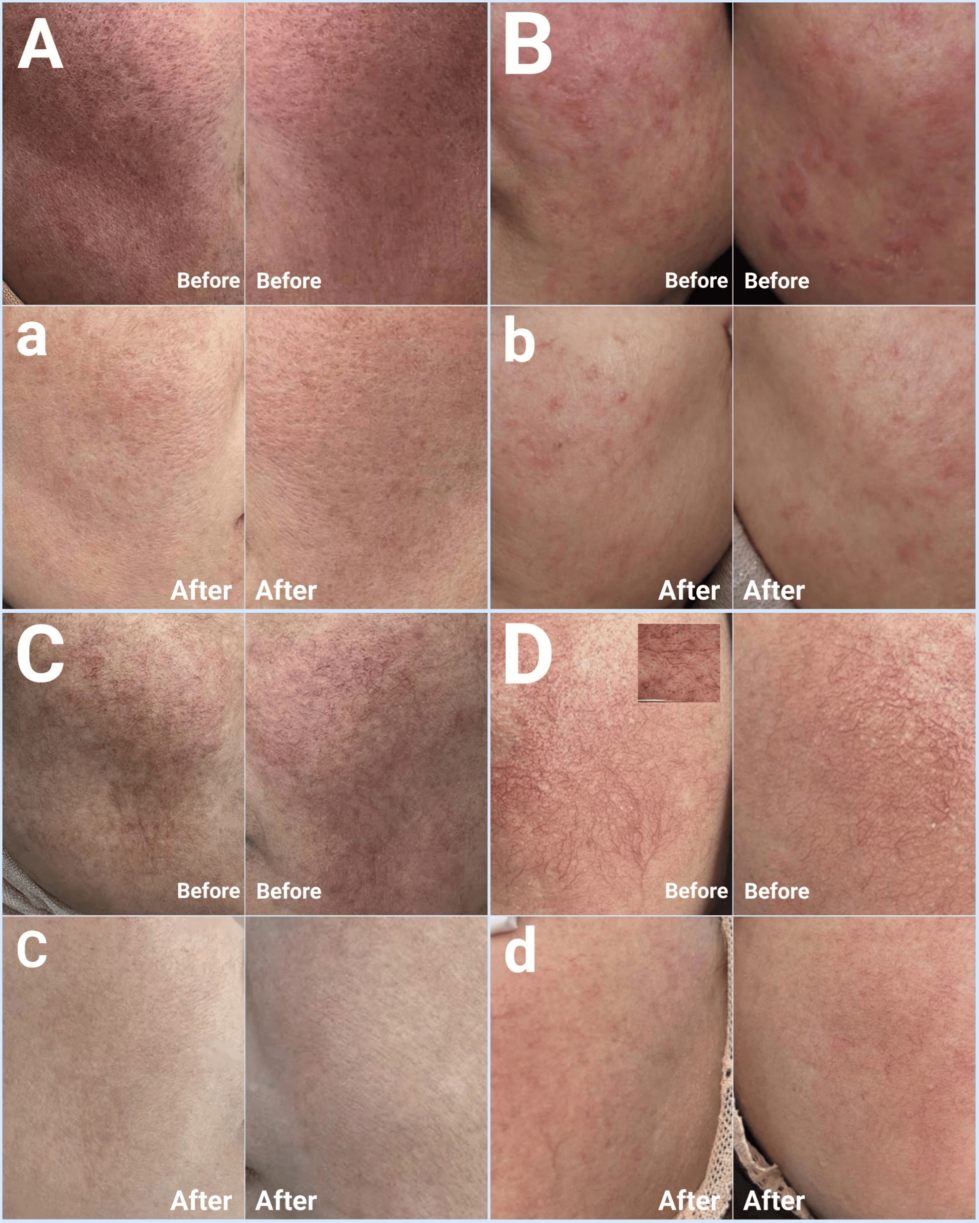
Representative clinical responses to 577-nm yellow laser therapy in steroid-induced rosacea. (A) Erythematous subtype before treatment and (a) very good response after three treatment sessions. (B) Papulopustular subtype before treatment and (b) very good response after three treatment sessions. (C) Erythematotelangiectatic subtype with network-like vessels before treatment and (c) very good response after three treatment sessions. (D) Erythematotelangiectatic subtype with arborizing superficial capillaries (dermoscopic inset) before treatment and (d) very good response after four treatment sessions. Source: Author's own clinical photographs obtained during the study period. Images are published with written informed consent from the patient.

**Figure 5:**
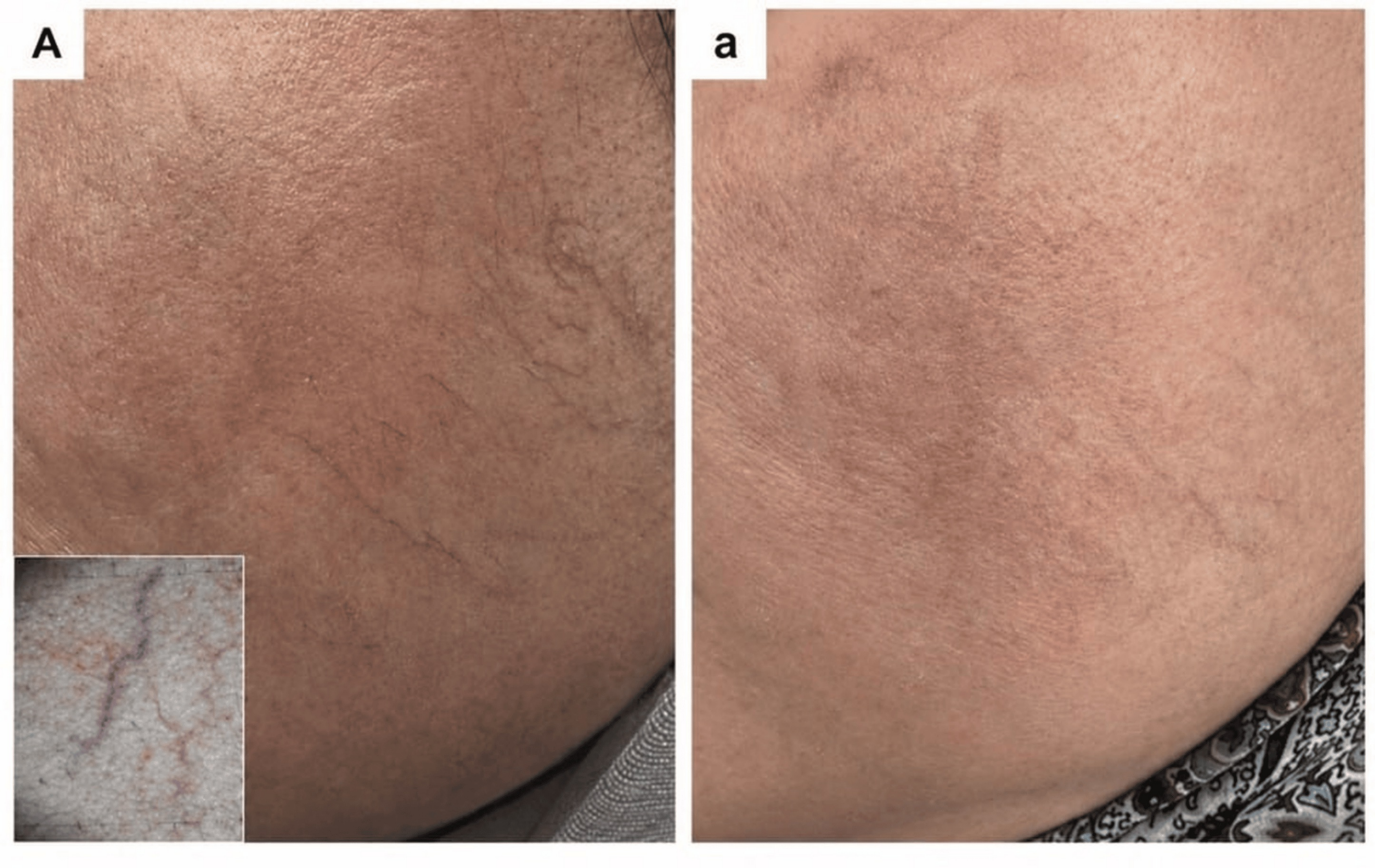
Clinical response of deep blue venules to 577-nm yellow laser therapy in steroid-induced rosacea. (A) Deep blue venules before treatment, with a dermoscopic inset demonstrating a prominent blue vascular channel. (a) Follow-up after treatment showing only moderate improvement, consistent with the relatively limited therapeutic response observed in this telangiectatic subtype. Source: Author’s own clinical photographs obtained during the study period. Images are published with written informed consent from the patient.

Likewise, no statistically significant association was observed among the major clinical subtypes (erythematous, papulopustular, and erythematotelangiectatic rosacea). However, within the erythematotelangiectatic group, the network-like telangiectatic subtype demonstrated the most favorable therapeutic response, with 12 of 28 patients (42.9%) achieving a very good response, whereas all patients with deep blue venules demonstrated only moderate improvement (Table 5; Figures 3B, 4, 5).

**Table 5:** Treatment response of telangiectatic subtypes to 577-nm yellow laser therapy. Response-category values are n (%) calculated within each subtype’s own total. The % column (after Total) denotes each subtype’s proportion of all patients with a classifiable telangiectatic morphology (n = 42); the three patterns were mutually exclusive and sum to 100%. Response categories: moderate (20-49%), good (50-74%), very good (≥75% improvement). P-values were obtained using the test specified in the Statistical analysis section.

Telangiectatic subtype	Total (n)	%	Moderate n (%)	Good n (%)	Very good n (%)	P-value
Network-like vessels	28	66.7	3 (10.7)	13 (46.4)	12 (42.9)	0.014
Arborizing superficial capillaries	10	23.8	4 (40.0)	6 (60.0)	0 (0.0)	0.014
Deep blue venules	4	9.5	4 (100.0)	0 (0.0)	0 (0.0)	<0.001

Side effects

Treatment was generally well tolerated, and no serious adverse events were reported. Most adverse effects were mild and transient, including self-limited erythema that resolved within 48 hours and mild treatment-related irritation.

The most common adverse effect was facial edema, which occurred in all patients and resolved within two to seven days. Post-inflammatory hyperpigmentation developed in two patients (4%) and resolved completely within two to three months without additional intervention.

## Discussion

The management of SIR remains clinically challenging, requiring corticosteroid withdrawal, trigger avoidance, and supportive medical therapy [3]. Recalcitrant cases may require adjunctive therapies; Seok et al. reported successful treatment of steroid-induced rosacea using topical tacrolimus combined with a 595-nm pulsed dye laser [9]. Likewise, Maria et al. demonstrated significant improvement in erythema and telangiectasia following intense pulsed light (IPL) therapy in patients with topical steroid-dependent facial dermatitis [10]. Epidemiologic studies from different geographic regions have further highlighted the clinical burden and demographic characteristics of steroid-induced rosacea [11]. In addition, concerns regarding inappropriate corticosteroid-containing skin-lightening preparations have been emphasized as an important public health issue [12]. Several emerging therapeutic modalities, including platelet-rich plasma (PRP) and topical tranexamic acid, have demonstrated encouraging results; however, evidence remains limited and largely derived from small studies [13,14]. Laser- and light-based therapies have therefore emerged as attractive treatment options for persistent erythema and telangiectasia.

Previous studies evaluating vascular laser therapy in rosacea have reported favorable outcomes. Seo et al. compared dual-wavelength long-pulsed 755-nm alexandrite/1064-nm neodymium-doped yttrium aluminum garnet (Nd:YAG) laser therapy with 585-nm pulsed dye laser treatment and demonstrated comparable reductions in erythema and patient satisfaction [15]. Additional comparative studies have demonstrated the efficacy of nonpurpuragenic pulsed dye laser, potassium titanyl phosphate (KTP) laser, IPL, and Nd:YAG laser systems in the treatment of facial erythema and telangiectasia [16-18].

The 577-nm yellow laser has previously demonstrated efficacy in rosacea, facial erythema, and telangiectasia because of its selective absorption by oxyhemoglobin and favorable safety profile [4,6,7]. However, its role in steroid-induced rosacea has not previously been investigated. To the best of our knowledge, the present study represents the first prospective evaluation of 577-nm yellow laser therapy specifically in patients with SIR.

Our findings demonstrated significant clinical improvement in both symptoms and clinical signs following the standardized treatment protocol, which combined topical corticosteroid withdrawal with 577-nm yellow laser therapy. Although the absence of a control group precludes definitive attribution of these improvements exclusively to laser treatment, the favorable outcomes support the potential role of 577-nm yellow laser as an adjunctive therapeutic modality in the management of steroid-induced rosacea. The most pronounced responses were observed for erythema, flushing, and telangiectasia, supporting the concept that the primary therapeutic target of the 577-nm wavelength is the abnormal superficial vasculature characteristic of steroid-induced rosacea. In contrast, xerosis, dryness, wrinkles, and comedones exhibited comparatively limited improvement, suggesting that these manifestations are less dependent on vascular pathology.

A novel finding of the present study was the differential response observed among telangiectatic subtypes. Network-like and arborizing superficial vessels appeared to demonstrate more favorable clinical responses than deep blue venules; however, this observation should be interpreted cautiously because the deep blue venule subgroup was small. This observation highlights the importance of dermoscopic vascular morphology as a potential predictor of treatment response. The relatively poor response of deep blue venules may be explained by their larger diameter and deeper dermal location, which are less optimally targeted by the penetration depth and absorption characteristics of the 577-nm wavelength [19].

Interestingly, treatment response was not significantly associated with age, sex, corticosteroid type, number of sessions, or major clinical subtype. Although no statistically significant associations were identified between overall treatment outcome and the evaluated demographic or clinical variables, these findings should be interpreted cautiously because of the limited sample size, particularly within certain subgroups.

The predominance of female patients in the present study is consistent with previous reports of steroid-induced rosacea [1,8,11]. This observation may reflect the higher prevalence of melasma and cosmetic use of topical corticosteroids among women, particularly through unregulated skin-lightening preparations commonly known as Khalta in Iraq. Similar concerns regarding inappropriate corticosteroid-containing skin-lightening products have been highlighted in international studies [12].

The prevalence of telangiectasia in our cohort (92%) was higher than that reported in previous studies [1,3,8,11]. This difference may be attributed to the routine use of dermoscopy, which enabled detailed characterization of vascular morphology and identification of subtle network-like vessels that might otherwise be classified as diffuse erythema during naked-eye examination.

An additional observation was the improvement of steroid-associated facial hirsutism following treatment. Although the mechanism underlying this finding remains unclear, a reduction in hirsutism was documented in most affected patients and may warrant further investigation in future studies.

Treatment was generally well tolerated. The most common adverse effect was transient facial edema, which resolved spontaneously within several days. Post-inflammatory hyperpigmentation occurred in only two patients and resolved completely during follow-up. No permanent adverse effects or treatment discontinuations were recorded.

Limitations

Several limitations should be acknowledged. First, the study was conducted at a single center and included a relatively small sample size. Second, the predominance of female participants (96%) may limit the generalizability of the findings to male patients. Third, Fitzpatrick skin phototypes V-VI were excluded; therefore, the safety and efficacy of the 577-nm yellow laser in darker skin types require further investigation. Fourth, laser treatment was initiated concurrently with a standardized corticosteroid tapering protocol. Consequently, the independent contribution of laser therapy cannot be distinguished from the natural clinical improvement that may occur following corticosteroid withdrawal alone. Therefore, the observed outcomes should be interpreted as reflecting the combined treatment approach rather than a direct causal effect of laser therapy.

Finally, the relatively short follow-up period of one month after the final treatment session precludes assessment of the long-term durability of treatment response and recurrence rates.

## Conclusions

The findings of this prospective study suggest that 577-nm yellow laser may represent a safe and promising adjunctive treatment for steroid-induced rosacea, particularly for improving erythema, flushing, and superficial telangiectasia when used as part of a standardized treatment protocol. Beyond demonstrating favorable short-term clinical outcomes, this study highlights the potential value of dermoscopic vascular morphology in predicting treatment response, with network-like telangiectasia showing the most favorable outcomes. Given the absence of a control group, the relatively small sample size, and the short follow-up period, larger controlled studies with longer follow-up are warranted to validate these findings and further define the role of 577-nm yellow laser therapy in the management of steroid-induced rosacea.
